# The Microstructure-Mechanical Properties of Hybrid Fibres-Reinforced Self-Compacting Lightweight Concrete with Perlite Aggregate

**DOI:** 10.3390/ma11071093

**Published:** 2018-06-27

**Authors:** Danuta Barnat-Hunek, Jacek Góra, Wojciech Andrzejuk, Grzegorz Łagód

**Affiliations:** 1 Faculty of Civil Engineering and Architecture, Lublin University of Technology, Nadbystrzycka Str. 40, 20-618 Lublin, Poland; d.barnat-hunek@pollub.pl (D.B.-H.); j.gora@pollub.pl (J.G.); 2 Faculty of Economic and Technical Science, Pope John Paul II State School of Higher Education in Biała Podlaska, Sidorska Str. 95/97, 21-500 Biała Podlaska, Poland; w.andrzejuk@dydaktyka.pswbp.pl; 3 Faculty of Environmental Engineering, Lublin University of Technology, Nadbystrzycka Str. 40B, 20-618 Lublin, Poland

**Keywords:** self-compacting concrete, basalt fibre, steel fibre, perlite, frost resistance, microstructure-mechanical properties, interfacial transition zone

## Abstract

The purpose of this paper is to determine the influence of the lightweight porous perlite aggregate and two widely used types of fibres on the physical and mechanical properties, frost durability and microstructure of self-compacting lightweight concrete (SCLC). The experimental investigation consisted of tests carried out on cubes and prismatic samples made of SCLC and fibres-reinforced SCLC with variable content ranging from 0.5 to 1% of basalt fibres (BF) and/or 0.5% of steel fibres (SF). In this study, two variable contents of fine perlite aggregate were used: 5% and 15%. The workability (the slump-flow and *t*_500_ values) in fresh state SCLCs have been done. Extensive data on compressive and flexural tensile strength in bending behaviour, frost resistance and the microstructure including interfacial transition zone (ITZ) were recorded and analysed. The hybrid fibres-reinforced SCLC with perlite aggregate showed a more ductile behaviour compared to that of SCLC without fibres. Fibres bridge cracks during flexural tensile strength test. BF successfully protected porous SCLC against frost attack, whereas SF succumbed to damage.

## 1. Introduction

In recent years, there has been a growing interest in self-compacting concrete (SCC), as shown by an increasing number of studies on this topic [1,2,3,4,5]. SCC is characterised by a high resistance to segregation and high flowability, enabling it to fill the mould and encapsulate the reinforcing elements entirely, without mechanical compaction [4,6,7]. The guidelines for the SCC design were determined, including: Reducing the water/binder ration, increasing the volume of cement paste, controlling the total volume of coarse-grained aggregate, controlling its maximum grain size and applying high-quality superplasticiser with numerous admixtures modifying viscosity in order to ensure balance between formability and stability [6,7,8,9]. A self-compacting concrete mixture should be characterised by a relatively low yield strength, average viscosity providing appropriate resistance to segregation and bleeding, as well as sufficiently stable workability during transportation and concreting. In comparison with normal concrete, SCC exhibits numerous advantages, including reduction of the construction time, labour, equipment use and noise at construction sites, because the need for mixture vibration is eliminated [8].

SCC containing lightweight aggregates (LWA) have also gained increasing popularity, e.g., Pollytag. LWA allows for interior curing based on the gradual release of water from presaturated LWA balancing interior moisture content, which was described by Kaszyńska, M. and Zieliński, A. [10,11,12]. SCLC uses recycled lightweight aggregates such as rubber granules [13], lightweight expanded clay aggregates [14], sugarcane bagasse ash [4], oil-palm-boiler clinker (OPBC)—a solid waste from the oil palm industry [15], pumice, volcanic tuff and diatomite [16], recycled modified polypropylene (PP) plastic particles [17]. Yang, S. et al. observed that the slump flow value is improved with an increase in the plastic content and the slump loss is reduced. When the percentage of sand replacement with plastic is relatively low (up to 20%), the infiltration through concrete is improved. The viscosity of SCL is reduced as the sand replacement level increases up to 15% [17].

Application of various types of fibres is recommended in SCC, which was discussed by Aslani, F. [18,19,20]. Fibres are added to the matrix as a reinforcement to limit cracking and improve the total ductility of the material [21,22]. Literature indicates that due to their properties, basalt fibres (BF) are applied in concretes to reinforce cement matrices; these include high durability, better stability, improved strength, and resistance to repeated impacts [4]. Basalt fibres in mortars improve the flexural and tensile strength, ductility, as well as cracking energy of cement materials [21,22]. Ralegaonkar, R. et al. indicate that the efficient basalt fibres addition approximates 1–3% (*w*/*w*) of binder [22]. Addition of basalt fibres to the mortar reduces the shrinkage during drying as well as significantly improving the abrasion, frost, and alkali resistance [23,24]. The fibres frequently increase porosity and absorptivity, which was indicated by the authors in other works [25,26]. Another type of fibres that are widely employed in diverse applications is the steel fibre (SF). Numerous studies reported that SF may successfully mitigate cracking and the occurrence of scratches in concrete and thus in buildings [21,27,28]. The presence of SF has an insignificant influence on the compressive strength of concrete; it improves the flexural and tensile strength as well as the dynamic modulus of elasticity [25,27]. The frost resistance of concrete with SF is relatively low, as indicated by Smarzewski, P. and Barnat-Hunek, D. [27]; it also depends on its porosity, aggregate and fibre properties as well as environmental conditions. Gao, D. et al. [29] mentioned that the surface of steel fibre should be covered with mortar to ensure adequate quality of concrete reinforced with steel fibres against corrosion factors.

The resistance of concrete to the freezing-thawing (F-T) phenomenon depends on the degree of saturation with water, layout of pores in hardened cement paste, and the type of aggregate used. Low permeability and low water/binder ratio constitute the basic properties characterizing the concrete with high freezing-thawing resistance [30]. As far as the cement-based composites are concerned, few studies on the behaviour during freezing and thawing for fibre-reinforced concrete can be found [21,27,30]. This is especially true in the case of hybrid fibres-reinforced SCLS; to the best of our knowledge, no research has been conducted on this topic.

Generally, the cracks in concrete appear for a number of reasons, including shrinkage in hardened concrete [31] or frost attacks [32]. These cracks weaken the waterproofing properties of concrete and expose its microstructure to harmful factors and substances, including moisture, chlorides and sulphites [33]. Cracks appear more frequently in lightweight concretes due to their lower strength and frost resistance rather than normal or high-performance concretes [34]. These cracks appear in different sizes and at different stages of concrete floor exploitation, including the ones made with self-compacting concrete; therefore, application of fibres with diversified length and quality is a good solution to this issue. The main aim of combining different types of fibres is to control cracks of various sizes in different concrete zones [27,35,36,37].

To the best of our knowledge, hybrid fibres-reinforced SCC with a lightweight aggregate such as perlite, have not been tested in terms of microstructure and its influence on wettability, strength and frost resistance of these special concretes. Therefore, these factors were determined using a special for microstructure-properties relationship. The scanning microscopy study of microstructure hybrid fibres-reinforced SCC with lightweight perlite aggregate aimed to indicate a diversified geometrical microstructure of the considered SCCs containing BF and SF, which has a significant influence on their physical and mechanical properties, especially frost resistance. Self-compacting concrete with a simultaneous application of perlite and mixture of fibres may be employed in the production of the construction elements in which its properties are desirable, e.g., for the production of self-compacting industrial flooring, load-bearing concrete slabs.

## 2. Materials and Methods

### 2.1. Aim and Scope of the Experiment

The aim of the work was to investigate and evaluate the self-compacting properties of perlite concrete with the addition of BF and SF. For the sake of comparison, the same experiments were conducted for the concretes without fibres. In order to expand the scope of evaluation, the concretes with different perlite content (5% and 15%) were investigated. The mechanical properties of SCLC and the properties responsible for the durability of structures under harsh climatic conditions were studied and the analysis of their microstructure was carried out.

### 2.2. Materials

The concrete was prepared using CEM I 42.5 R Portland cement. The chemical composition of cement was presented in Table 1, whereas the physical properties were shown in Table 2 [38]. The optimal value of water/binder ratio and the content of self-compacting concrete were determined experimentally, by preparing multiple test batches.

Washed quartz sand, 2–8 mm granite aggregate, perlite (5% and 15% sand replacement) and micro-silica were applied for the preparation of mixtures. Six concrete mixtures were prepared with the composition presented in Table 3 (water/binder ratio was calculated as water/(cement + k silica fume)). Steel fibres (SF) and basalt fibres (BF) were not added in two mixtures—C5P0, C15P0; 1% BF admixture was utilised in C5P1b and C15P1b mixtures, whereas a 0.5% SF and BF addition was employed in C5P50b50s and C15P50b50s. 

Perlite (0/2 mm), hydrated acidic sodium-potassium aluminosilicate with the density of 95 kg·m^−3^ also contained different components. It was mainly consisting of silica SiO_2_ (65–75%), aluminium, sodium, potassium, magnesium, calcium and iron oxides [Al_2_O_3_ (10–18%), K_2_O + Na_2_O (6–9%), MgO + CaO (2–6%) and Fe_2_O_3_ (1–5%)]. Water absorptivity of perlite ranges from 3 to 5% [39]. Granite aggregate (2–8 mm) was characterised by the following properties: Apparent density—2650 kg·m^−3^, open porosity—0.95%, water absorption—0.35%, flexural strength—11.5 MPa, compressive strength—190 MPa, and abrasion resistance—6050 mm^3^/5000 mm^2^ [40]. The utilised SF were characterised by the following parameters: 50 mm in length, 1000 µm in diameter, 7800 kg·m^−3^, tensile strength of 1100 MPa and elastic modulus of 200 GPa [25,27]. The silica fume in the form of powder had a specific surface of 15–30 m^2^·g^−1^ [27]. Silica fume comprises spherical molecules which are much smaller than the ones of cement. The increased surface area makes it highly reactive and may ensure high early strength gain, low concrete permeability, and reduce the probability of bleeding to occur [13]. The basic components of commercial silica fume used in this study were as follows: SiO_2_—96%, CaO—1.5% by weight, specific gravity 2170 kg·m^−3^ [41]. Quartz sand (0/2 mm) has specific gravity of 2650 kg·m^−3^ and water absorption of 1.2%, and moisture content of 0.16% [42]. The parameters of applied BF are presented in Table 4 [26,43].

The used BF had the length of about 24 mm and low diameter of 11–18 µm. The minerals of the considered basalt rock include plagioclase: Na (AlSi_3_O_8_)-Ca (Al_2_SiO_8_); pyroxene: XY_2_[(Si,Al)_2_O_6_] (where X–Ca, Mg, Fe^2+^ and Y stands for Fe^3+^, Al, Ti); and olivine: (Fe,Mg)_2_ SiO_4_ which was proven by Kamiya, S. et al. [44] in a US Patent.

In order to obtain the same workability in all the mixtures, an efficient superplasticiser (Sika Visco Crete 20 HE) based on polycarboxylate ethers, was added in the amount of 2% in relation to the weight of cement and silica fume. The amount of admixture was determined experimentally. The admixture met the requirements of superplasticisers specified in EN 934-2 + A1:2012 standard [45]. The parameters of applied superplasticisers are: density of 1.08 g·cm^−3^ at 20 °C, pH 4.5; chloride content <0.10%, alkali content <0.8 [46].

### 2.3. Methods

All components except for BF and SF were first mixed for 2 min; afterwards, BF and/or SF were added. Following another two minutes, water was added and mixed for subsequent 2 min.

The concrete mixtures were investigated first; then, moulds were filled to the half of their capacity and compacted for 1 min on a vibrating table. Afterwards, a second layer of mixtures was laid and the samples were compacted again. The samples were stored under laboratory conditions for 24 h and then removed from the mould and stored in water at the temperature of 20 ± 2 °C for 7 and 28 days.

#### 2.3.1. Research Characteristics of Concrete Mixtures

In accordance with the PN-EN 12350-8:2012 standard [47], the workability was analysed with a slump-flow test and the assessment of viscosity class *t*_500_. An Abrams cone was placed in the middle of a stainless-steel plate with a flat and smooth surface and the dimensions of 900 mm × 900 mm. Then, the cone was filled in such a way so that the concrete did not leak from the bottom, without mixing and compacting. The excess on the top was evened out. After 30 s at most, the cone was forcefully lifted upwards (1–3 s). Following the moment of cone separation from the plate, time *t*_500_ was measured with the accuracy of 0.1 s until the mixture achieved the diameter of 500 mm. Afterwards, the diameter of flow was measured in two perpendicular dimensions, rounded up to 10 mm. The results of slump-flow measurement were determined using formula (1) with the accuracy of up to 100 mm [47].

(1) $$SF=\frac{d_{1}\;+\;d_{2}}{2}$$

Time *t*_500_ was provided with the accuracy up to 0.5 s. The consistency class was determined with the slump-flow test and the viscosity class *t*_500_ was specified on the basis of Table 5 and Table 6, respectively. 

Assessment of concrete mixture stability was performed after the slump-flow test. It involved checking whether the circumference of mixture patch showed visible segregation in the form of slurry film or water as well as observing even distribution of the aggregate. Attention was drawn to whether the coarse-grained aggregate accumulated in the middle of the mixture patch.

The Visual Stability Index (VSI) of self-compacting mixtures was determined on the basis of Table 7.

**Figure 1:**
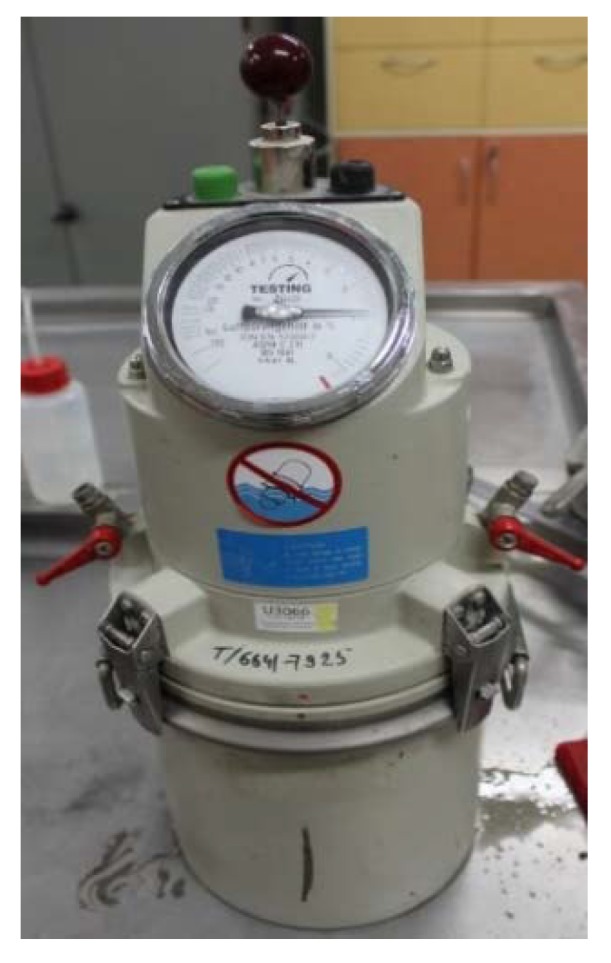
Measurement of the air content in a concrete mixture.

The air content in the concrete mixture was measured in line with PN-EN 12350-7 standard [49], using the pressure method. The device made by TESTING Bluhm & Feuerherdt (Berlin, Germany) was used for the investigation (Figure 1).

#### 2.3.2. Research Characteristics of Hardened Concrete

In order to determine the physical properties, 6 cubic samples with the edge length of 100 mm were prepared from each concrete. They were used for studying the volumetric density, frost resistance, porosity and water absorption by weight. As far as the mechanical properties are concerned, 12 cubic samples with the edge length of 100 mm were prepared for the compressive strength test (6 samples were examined following 28 days of maturation and the other 6 after 7 days of maturation), and another 12 cubic samples with the same dimensions were used for frost resistance test. The flexural tensile strength was tested with 6 cuboid samples with the dimensions of 100 mm × 100 mm × 500 mm.

**Figure 2:**
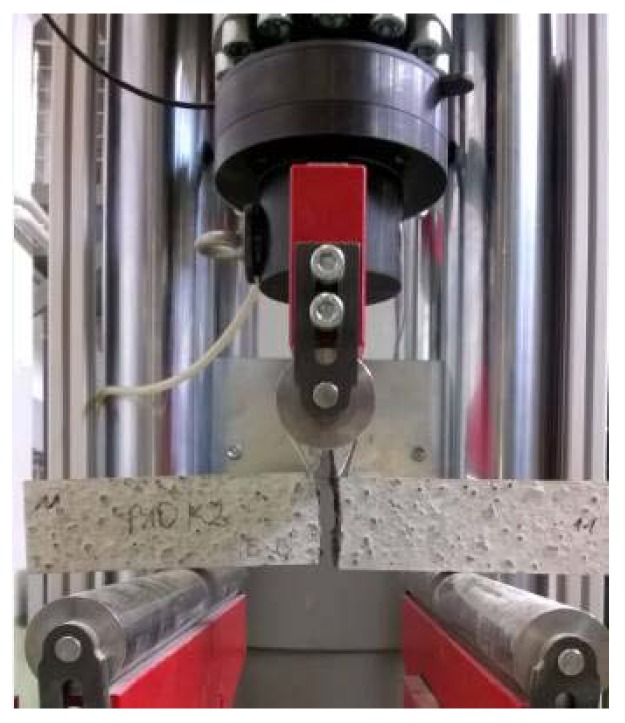
Flexural tensile strength test of C5P1b concrete.

**Figure 3:**
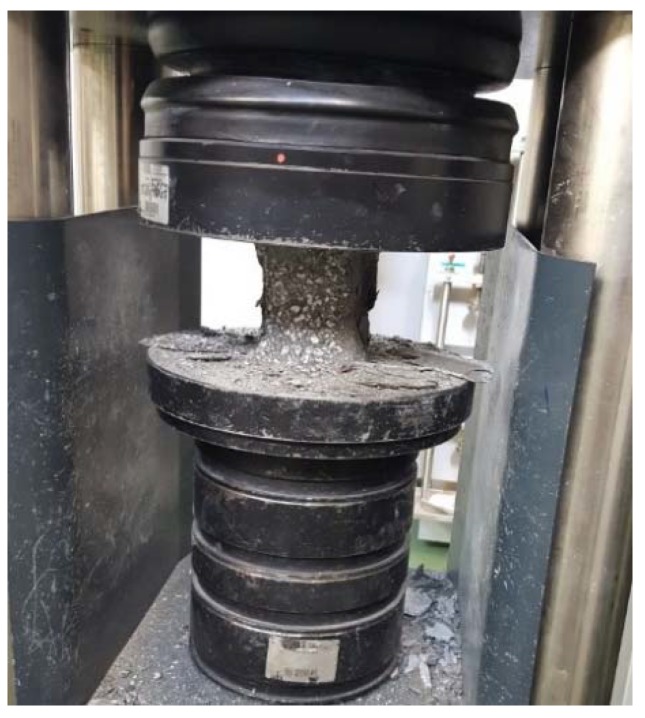
Compressive strength test of C15P1b concrete.

The samples used for the water absorption by weight test were placed on a 10 mm grate suspended over the bottom of a bathtub and submerged in water to ½ of their height. After 24 h, the water level was increased so that it reached 10 mm over the samples. Following another 24 h, the samples were removed, wiped and weighed. The concrete samples were saturated for so long that the subsequent two weightings showed no increase in mass. After saturation, the samples were dried to constant mass and weighed, then water absorption by weight and volumetric density of concrete were determined in accordance with the PN-B-06250:1988 standard [50]. The porosity of concretes was studied with Mercury Injection Capillary Pressure (MICP) method, using AutoPore IV 9520 (Micrometrics, GA, USA). The measurement method was described by Rigby SP. et al. [51] and Sidney D. [52]. It enables investigation of pores with diameters ranging from 3 nm to 200 μm. The surface of the sample approximated 1 cm^2^ and it was dry, because the presence of other liquids prevents the injection of mercury. Cubic samples after 28 days of maturation were used for frost resistance test, which was carried out after saturation of samples with water, similarly to the water absorption tests. Freezing was conducted at the temperature of −20 °C for 4 h. Afterwards, the samples were thawed for 4 h in water heated to the temperature of +20 °C. The weight loss of samples and a decrease in compressive strength in relation to the reference samples kept in water at the temperature of +20 °C ± 2 °C throughout the experiment were evaluated, in line with PN-B-06250:1988 [50]. The flexural tensile strength (Figure 2) was tested on the cuboid samples conforming to the PN-EN 12390-5 standard [53]. The experiment involved placing the samples in a universal testing machine and conducting a three-point flex test. The maximum load was noted, and the flexural tensile strength was calculated afterwards. The experiment was conducted on each prepared concrete, after 28 days of maturation. The flexural tensile strength test was performed on cubic samples with the edge length of 100 mm, in accordance with the PN-EN 12390-3 standard [54] (Figure 3). The relationship between the compressive strength values of particular sample types was assumed in line with PN-B-06250:1988 [50] *f*_c,cube#150_ = 0.9 × *f*_c,cube#100_, where: *f*_c,cube#150_, *f*_c,cube#100_—compressive strength obtained on cubic samples with the edge length of 150 and 100 mm, respectively.

Scanning electron microscopy SEM (Quanta FEG 250 microscope by FEI, Hillsboro, OR, USA) was employed to determine the morphology and microstructure of concrete. The samples for SEM studies were glued to a carbon holder by means of carbon glue. Afterwards, they were covered with a 50 nm thick layer of carbon in a coating machine in order to achieve conductivity on the surface of the sample. The methodology of sample preparation excludes the possibility of micro-defects due to concrete surface and cracking. In order to avoid the possibility of other surface defects, low vacuum and low beam energy were applied.

## 3. Results

### 3.1. Properties of Concrete Mixture

The slump-flow test, time of distribution *t*_500_, mixture stability assessment according to VSI and the air content in mixtures were shown in Table 8.

### 3.2. Properties of Hybrid Fibres-Reinforced Self-Compacting Lightweight Concrete

Table 9 presents the results of physical and mechanical properties of the considered concretes.

**Figure 4:**
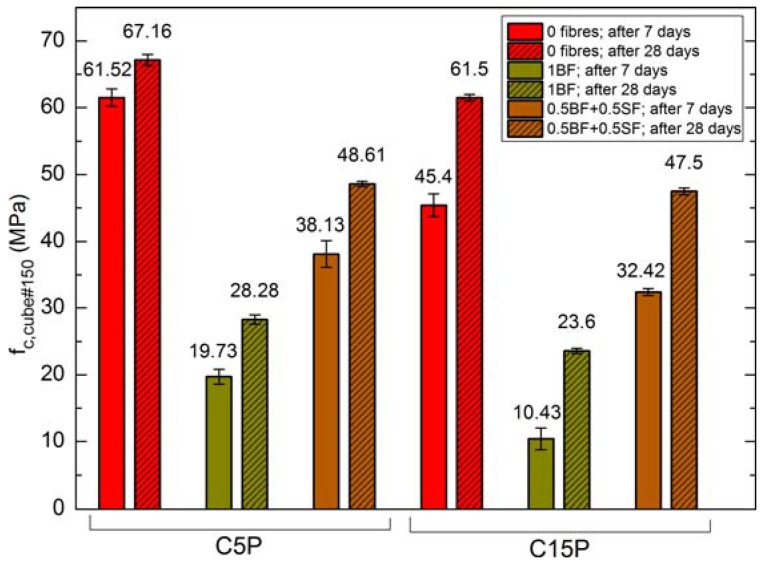
Compressive strength of SCLC after 7 and 28 days.

**Figure 5:**
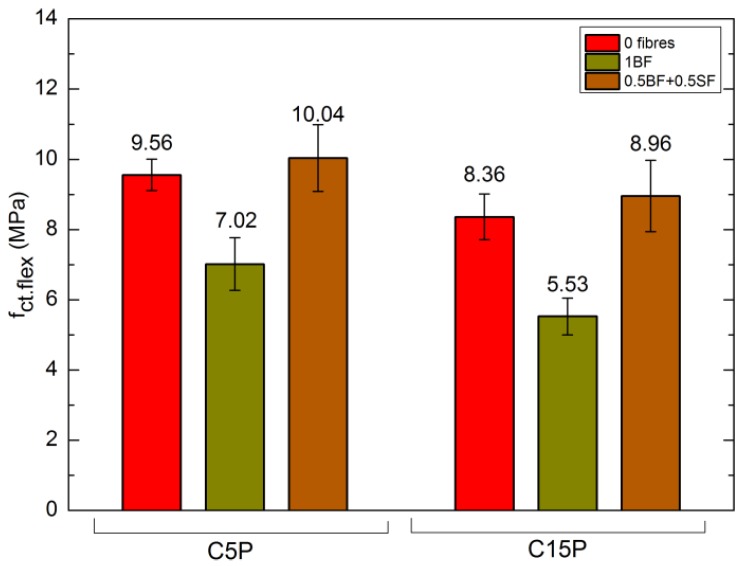
Flexural strength of SCLC after 28 days.

Figure 4 presents the compressive strength values of cubic samples with edge length of 150 mm after 7 and 28 days of maturation, whereas Figure 5 shows flexural strength after 28 days of maturation.

**Figure 6:**
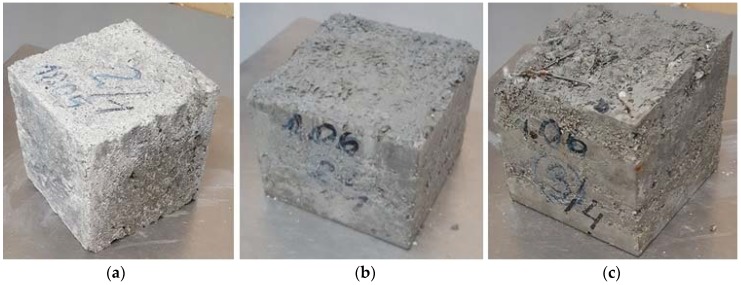
Condition of concrete following 50 F-T cycles: (**a**) C5P1b; (**b**) C15P0; (**c**) C15P50b50s.

Figure 6 presents the condition of selected, representative samples of SCLC following 50 F-T cycles.

### 3.3. Microstructure of Hybrid Fibres-Reinforced Self-Compacting Lightweight Concrete

**Figure 7:**
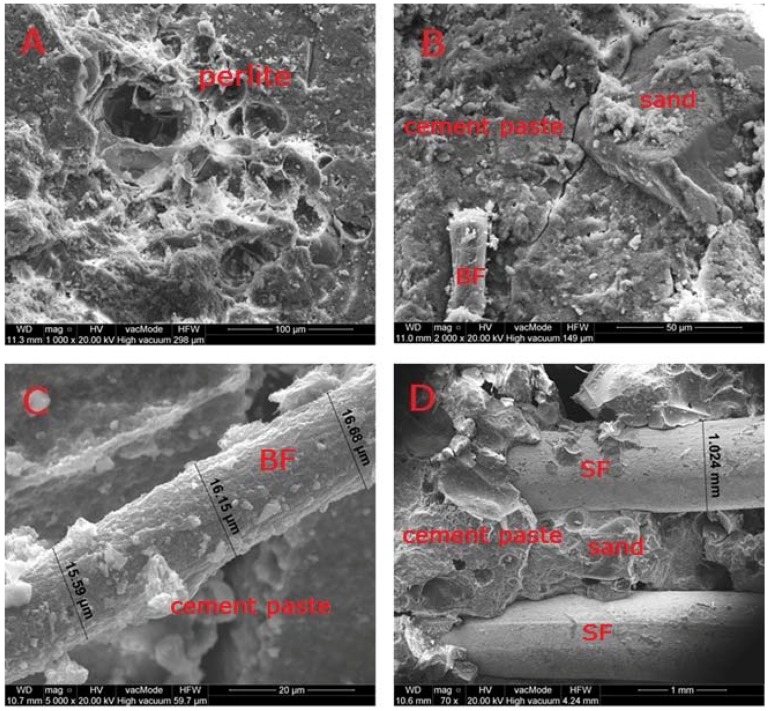

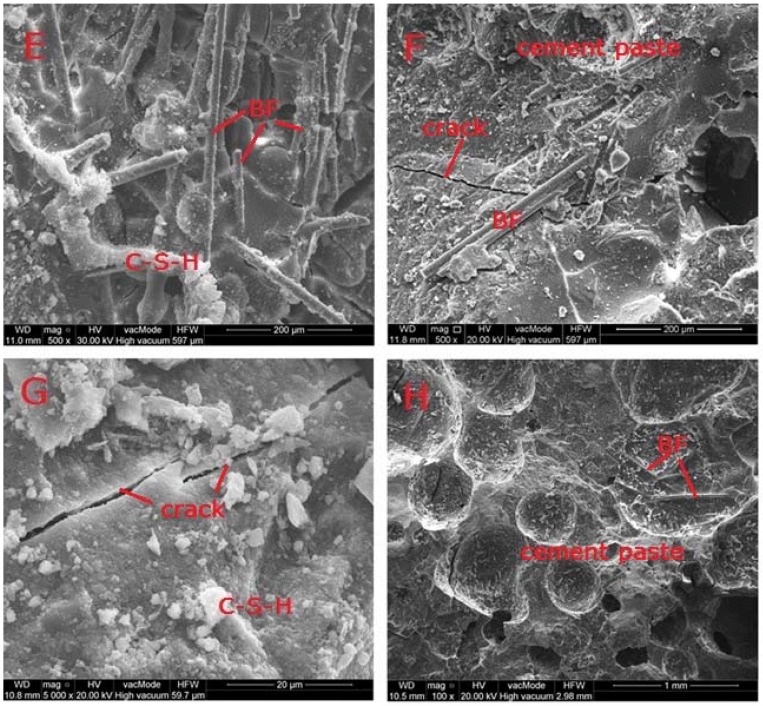
Microstructure of hybrid fibres-reinforced SCLC: (**A**) C15P0 (×1000); (**B**) C15P1b (×2000); (**C**) C5P1b (×5000); (**D**) C5P50b50s (×70); (**E**) C15P1b (×500); (**F**) C15P1b (×500); (**G**) C5P50b50s (×5000); (**H**) C15P1b (×100).

Figure 7 presents the microstructure and adhesion of cement paste to BF, SF and aggregates.

**Figure 8:**
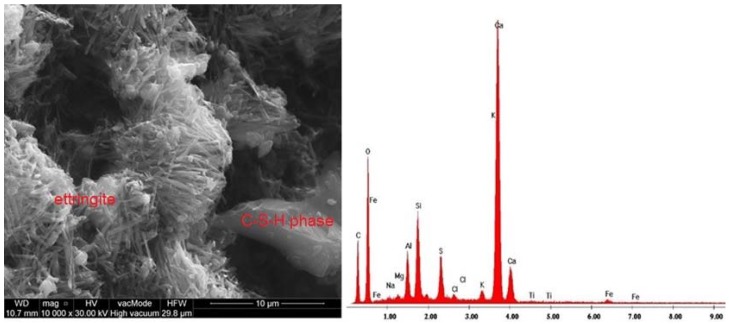
Microstructure of SCC C5P1b and EDS elemental analysis area rich in ettringite crystals between aggregated clusters of C-S-H phases (×10,000).

**Figure 9:**
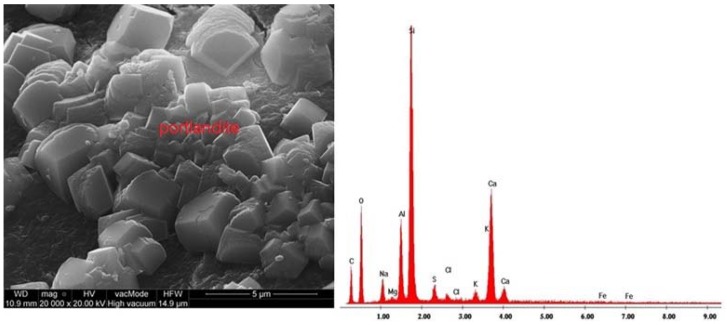
Microstructure of SCC C15P1b and EDS elemental analysis—area rich in crystals of portlandite (×20,000).

**Figure 10:**
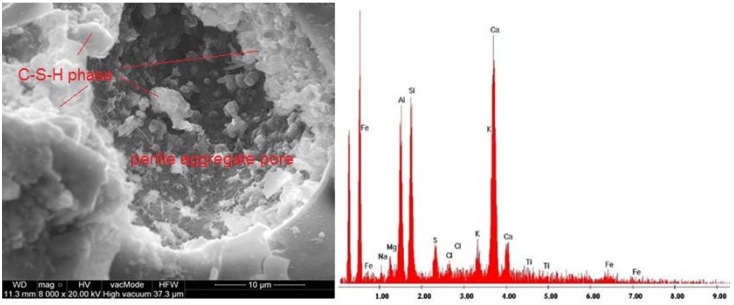
Microstructure of SCC C5P0 and EDS elemental analysis–perlite aggregate pores filled with C-S-H phase (×8000).

The SEM images of samples microstructure were supplemented with EDS graphs showing the chemical composition of the considered samples (Figure 8, Figure 9 and Figure 10).

## 4. Discussion

### 4.1. Properties of Concrete Mixtures

None of the studied mixes showed any signs of segregation, including bleeding. The value for SCLC without fibres amounts to 750 mm, whereas for the SCLC with 1% BF it equals 660 and 680 mm, which enabled to achieve SF2 required for SCC [13]. Not all values of slump-flow for the SCLC met the suggested range of values (600–700 mm). Mixtures with hybrid fibres achieved the SF1 class and the flow diameter of 550 to 600 mm. It was observed that the presence of fibres causes compaction of the mixture, resulting in a reduction of the SF class. Fibres, especially steel ones which are characterised by significant length and thickness, hinder the mixture distribution. This is mainly because the SF used in the test are of elongated shape, which may negatively influence the fluidity of fresh SCLC, lengthening the flow time *t*_500_ more than twice. Table 8 indicates that the viscosity is increased as the sand substitution changed from 5% to 15%; BF was added afterwards and then BF and SF were added simultaneously. The highest viscosity and the lowest SF_max_ are exhibited by the mixtures with hybrid fibres (C5P50b50s and C15P50b50s). The air content in these mixtures is also the highest, amounting to 6.0 and 6.2%, respectively, whereas for the mixture without fibres and lowest perlite content (C5P0), it only reaches 2.6%. As indicated in the course of research, fibres hinder the self-compaction of the mixture and its deaeration. Increasing the dose of superplasticiser should be considered in order to mitigate the drop in workability and ensure sufficient flow. However, caution is advised, because the mixtures with lightweight, porous aggregate are susceptible to segregation due to an increased amount of superplasticiser and more porous nature of lightweight aggregates, which was confirmed in the studies by Aslani, F. et al. [17] and Bandi, S.M. et al. [55].

### 4.2. Properties of Hybrid Fibres-Reinforced Self-Compacting Lightweight Concrete

On the basis of volumetric density, it was proven that only the C5P0 concrete should not be classified as a lightweight concrete (in accordance with the standard [47], the volumetric density of lightweight aggregate should not exceed 2000 kg/m^3^). This results both from the lowest air content in the mixture, as well the application of a single additive (perlite) in the amount lower than 5%.

The results on water absorptivity indicate a clear influence both of the porous perlite addition—the achieved values are significantly higher than the ones for normal concrete—as well as the increased porosity of mixtures due to the application of fibres, especially steel ones.

Generally, while evaluating the results of compressive strength test in the concretes containing 15% of perlite, lower values were obtained, regardless of the type of other applied additives. This especially concerns the early strength values after 7 days of maturation. The differences between the compressive strength values of C5P and C15P concretes decrease after 28 days of maturation (Figure 4). Following 7 days of maturation in C5P concrete, the 1% addition BF caused a reduction in strength by 68% whereas in the case of BF and BS—by 37% in relation to the strength of C5P0. The strength of C15P0 concrete was reduced by 77% and 29%, respectively. The observed reduction in the strength of C15P concrete are connected with the weakening of concrete structure caused by increased addition of porous perlite characterised by poor strength parameters.

Lower strength of fibre-reinforced concretes should be connected with twice as high air content in these mixture, as well as greater open porosity in the hardened concretes. The lowest strength values obtained in the concretes with the addition of BF are additionally connected with the Interfacial Transition Zone (ITZ) structure between the fibres and cement slurry. The ITZ between SF and cement slurry is significantly better. This was confirmed by the results of microstructural studies, which were described and explained in Section 4.3.

The influence of perlite and fibres addition on the flexural tensile strength is different. Increasing the perlite addition from 5% to 15% reduces the flexural as well as compressive strength of all concretes. On the other hand, considering the sole influence of BF and BS addition, the situation is different. The sole BF addition reduces the flexural strength by 27% in relation to C5P0 and by 34% compared to C15P0 (Figure 5). However, if a combination of fibres involving 0.5% BF + 0.5% BS is applied, then the flexural strength improves—by 5% in C5P50b50s and by 6% in C15P50b50s, in relation to C5P0 and C15P0, respectively.

The susceptibility of SCLC to the damage caused by freezing and thawing depends on the concrete composition, its absorptivity, porosity, as well as the content and the type of fibres. On the other hand, as far as the results of frost resistance test are concerned, it is difficult to indicate an unambiguous relationship between the mass loss and a reduction in strength. It seems that this parameter is influenced the most by the addition of perlite, which reduces the frost resistance. An improvement was achieved after the addition of BF, both with C5P1b and C15P1b. The application of combined SF and BF yielded no satisfactory effects. However, the only concrete that did not meet the standard requirements is C15P0, due to the mass loss equalling 7.88%, with the limit value of 5%, in line with the PN-B-06250:1988 standard [50]. The remaining results are within the permissible values.

Along with the increasing volume, the thawing water tends to relocate into voids. This process contributes to an increase in the hydraulic pressure, as reported by Fagerlund [56]. When the expansive force exceeds the tensile strength of concrete, microcracks begin appearing [57]. When the cracking process is initiated and the structure of SCLC with fibres is additionally cracked, as presented in Figure 6, a greater amount of water infiltrates into concrete and the damages caused by the freezing and thawing processes are more severe. The greatest mass loss (7.8%) and compressive strength reduction (8.1%) were observed in the P15P0 concrete without fibres and the highest amount of perlite (Figure 6b, Table 9). The content of lightweight aggregate, which reduces the tightness of concrete, could be the reason for the sample destruction.

In the case of the hybrid fibres-reinforced SCLC, as predicted, a significant concrete damage occurred when the amount of steel fibres increased during cyclic freezing and thawing. In the course of the experiment, numerous cracks and loosening in concrete as well the corrosion of SF appeared on the surface of C15P50b50s concrete samples (Figure 6c). In the concrete with SF, the increased number and volume of capillary pores and voids between steel and slurry is the main reason for expansive internal pressure during the freezing of water. Studies showed that steel fibres did not slow down the occurrence of microcracks and thus do not protect against failure during T-F cycles. Similar observations in UHPC concrete were described in paper [27]. The work by Afroughsabet, V., Ozbakkaloglu, T. [21] presented the positive influence of hybrid fibres after 100 cycles of concrete freezing and thawing of highly durable concrete with and without steel and polypropylene fibres. The flexural strength, compressive strength and dynamic modulus of elasticity values of concretes without fibres decreased by 23%, 14%, 9%, respectively, whereas in the case of the fibres-reinforced concretes, these parameters dropped by 12%, 10% and 8%.

In our studies, the basalt fibres successfully protected SCLS against the effect of frost, in contrast to SF. Despite an increased porosity and absorptivity of SCLC with BF, fine, thin fibres bridge scratches and cracks, protecting the concrete against frost damage (Figure 6a). The losses caused by frost are 70-fold smaller in the concrete with 5% perlite content and almost 6-fold smaller in the concrete with 15% perlite content (Table 9).

### 4.3. Microstructure of Hybrid Fibres-Reinforced Self-Compacting Lightweight Concrete

The microstructural analyses of SCLC were conducted on the samples taken from the undamaged parts of cuboids used for the tensile strength testing—the fragments which were not subjected to stresses. Figure 7 illustrates the interfacial transition zone (ITZ) between the cement paste and the grains of perlite (Figure 7A) and granite aggregate (Figure 7B) of hardened C15P1b concrete. ITZ is the weakest area of the material, in which microcracks are formed first; therefore ITZ has a decisive impact on the mechanical properties of concrete. The ITZ in lightweight concrete is different from the one in normal concrete, because perlite aggregate is a porous aggregate which intensively takes up water from cement paste [58]. The amount of water in the mixture has a great influence on the thickness of ITZ. Directly on the surface of granite aggregate, there is a layer of oriented portlandite crystals, as well as a C-S-H layer (Figure 7B). The factor determining the structure and ITZ properties in the analysed concretes is the adhesion between the rough perlite aggregate and cement matrix. A very good adhesion between the cement paste and perlite aggregate was observed (Figure 7A) in relation to the granite aggregate (Figure 7B). The bonding between granite and cement paste is weaker than in the case of perlite and cement paste, which is affected by the surface texture of crushed granite aggregate. The fracture of grains is glassy, conchoidal. This finding was confirmed by observations indicating 2–5 µm cracks between the granite aggregate and cement paste (Figure 7B). Figure 7C–F show the distribution and size of BF (Figure 7C,E,F) and SF (Figure 7D). Fibres are distributed randomly and their diameter ranges from 15 to 17 µm (BF) and 1.0–1.03 mm (SF). The cement paste has good bonding with the SF (Figure 7D), there were no micro-cracks or micro-fractures. However, there are spots where the bonding is absent and empty voids are visible between steel and cement paste. In the concretes with BF, a weak adhesion of cement paste to BF was observed. BF are characterised by a smooth, slick, hydrophobic surface that hinders bonding of these two materials (Figure 7C,E,F). If the content of slick BF is increased, a greater amount of free water around BF weakens the bonding of BF and cement paste, causing the occurrence of cracks, zones with voids and relatively weak adhesion, as presented in Figure 7. Similar observations were described in the paper by Yang, S. et al. [17]; however, they pertained to the adhesion of cement paste and slick surface of plastic aggregate. Nevertheless, this situation did not deteriorate the frost resistance. The adhesion of cement paste to BF is sufficient to ensure successful protection of SCLC against frost attack (Table 9).

In the case of concrete without fibres addition, there are no visible scratches or cracks and the structure is tight (Figure 7A). On the other hand, in SCLC with fibres, there are numerous cracks and air pores with sizes ranging from 0.1 to 0.55 mm (Figure 7E–H). The fibres-reinforced concretes are characterised with a greater porosity and absorptivity, as well as much lower compressive strength in relation to the concretes without fibres, which was confirmed in the studies performed by other authors, described in the paper (26). The authors showed that the addition of BF has a negative impact on the compressive strength and frost resistance of cement mortars. Along with the increase of BF, porosity increases by up to 32% [26]. In contrast to SCLC, the authors observed a very good adhesion of cement paste to BF and C-S-H phases have clearly crystallised in the cement mortars.

The hardened cement slurry prepared from Portland cement comprises about 70% of hydrated calcium silicates—C-S-H phases, approximately 20% calcium hydroxide as well as aluminate hydration products and calcium aluminoferrite. In the third hydration period, the pores of hardened cement paste are filled with short fibres or hydrated calcium silicate phases. The characteristic feature of this phase is the transformation of calcium aluminate trisulfate 3CaO·Al_2_O_3_·3CaSO_4_·32H_2_O to calcium aluminate monosulfate 3CaO·Al_2_O_3_·CaSO_4_·12H_2_O [59]. Ettringite crystals usually form elongated, rounded, needle-like crystals (Figure 8). On the other hand, Figure 9 shows a massive, lamellar structure of hexagonal crystals—Ca(OH)_2_ portlandite. Figure 10 presents the pores of perlite aggregate filled with C-S-H phase. Pores are rarely filled with ball type ettringite [60]. Ettringites were also observed by Ceesay, J. [61] and Dubberke, W. [62]. C-S-H phases are also visible in Figure 7B,C,E,G whereas portlandite lamella are shown in Figure 7G. Because the w/b ratio in the analysed SCLC is relatively high (Table 3), hydration of cement was not disrupted by porous, fine, water-absorbing lightweight aggregate, which was confirmed in the works by Kaszyńska, M. [10,11,12].

## 5. Conclusions

The research on the prospective application of SF and BF and perlite aggregate proved that they can be successfully utilised for the production of building materials such as SCLC.

A thorough analysis of the obtained results enables to formulate the following conclusions:

- Fibre-reinforced concretes, both with basalt and steel fibres, are characterised with greater porosity and absorptivity as well as much lower compressive strength in relation to the concretes without fibres addition. The lowest strength values were obtained for the concretes with BF addition. Microstructural studies showed that this is connected with the ITZ structure between fibres and cement slurry.
- Adding BF alone reduces the flexural strength by 27% in relation to C5P0 and by 34% compared to C15P0. However, if a combination of fibres involving 0.5% BF + 0.5% BS is applied, then the flexural strength improves—by 5% in C5P50b50s and by 6% in C15P50b50s, in relation to C5P0 and C15P0, respectively.
- As the addition of perlite increases, the absorptivity and frost resistance of considered concretes deteriorates. An improvement in frost resistance can be achieved by the application of BF in the amount of 1%. Utilising a combination of basalt and steel fibres no longer yields satisfactory results.
- The content of steel fibres significantly influences the increase in the air content within the concrete mixture, regardless of the perlite content. The air content in the mixture with steel and basalt fibres is 8% higher than in the mixture with basalt fibres, on average.
- The microstructural studies showed a much better ITZ structure of cement paste with perlite aggregate in relation to the granite aggregate. In turn, the ITZ between cement paste and fibres depends on their type. The cement paste exhibits good bonding with the steel fibres, there were no micro-cracks or micro-fractures. The ITZ between SF and cement slurry is significantly better than in the case of BF.
- The highest frost resistance was observed in the case of SCLC, which contain basalt fibres, rather than SF. Despite an increased porosity and absorptivity of SCLC with BF, thin fibres bridge cracks, protecting the concrete against frost damage; therefore, in the case of concrete intended for outdoor applications, the C5P1b concrete is recommended.
- In cases when concrete is to be applied indoors and the resistance to F-T cycles is not necessary, the SCLC concrete with the highest strength parameters, i.e., C5P50b50s is recommended.

## Figures and Tables

**Table 1:** Chemical composition and alkali content in cement clinker CEM I 42.5 R, %.

Compound	CaO	SiO_2_	Al_2_O_3_	Fe_2_O_3_	MgO	Na_2_O	K_2_O	Na_2_O_eq_
Content	64.64	20.22	4.37	3.33	1.20	0.26	0.50	0.59

**Table 2:** Physical properties of cement CEM I 42.5 R.

Le Chatelier (mm)	Spec. Surface (cm^2^·g^−1^)	Spec. Gravity (kg·dm^−3^)	Initial Setting Time (min)	Heat of Hydr. (J·g^−1^) *	2-Day Compr. Strength (MPa)	28-Day Compr. Strength (MPa)
1.1	4110	3.09	170	308	29.9	59.9

* Heat of hydration measured after 41 h with the use of semiadiabatic method.

**Table 3:** Composition of concrete mixtures.

Components	Unit	C5P0	C5P1b	C5P50b50s	C15P0	C15P1b	C15P50b50s
Portland cement CEM I 42.5 R	(kg·m^−3^)	461	461	461	461	461	461
Silica fume	(kg·m^−3^)	40	40	40	40	40	40
Granite aggregate (2/8 mm)	(kg·m^−3^)	936	936	936	936	936	936
Quartz sand (0/2 mm)	(kg·m^−3^)	664	637	637	594	567	567
Perlite (0/2 mm)	(kg·m^−3^)	1.25	1.25	1.25	3.76	3.76	3.76
Perlite (0/2 mm)	(%)	5	5	5	15	15	15
Water	(l·m^−3^)	208.4	208.2	208.2	208.2	208.2	208.2
Superplasticiser	(kg·m^−3^)	7.60	7.91	8.35	7.82	8.10	8.78
Steel fibres (SF)	(kg·m^−3^)	-	-	39.25	-	-	39.25
Steel fibres (SF)	(%)	-	-	0.5	-	-	0.5
Basalt fibres (BF)	(kg·m^−3^)	-	26.7	13.35	-	26.7	13.35
Basalt fibres (BF)	(%)	-	1	0.5	-	1	0.5
w/c	-	0.45	0.45	0.45	0.45	0.45	0.45
w/b	-	0.41	0.41	0.41	0.41	0.41	0.41

**Table 4:** Parameters of basalt fibres [26,43].

Parameters	Unit	Value	Parameters	Unit	Value
Hardness on the Mohs scale	(−)	8.5	Moisture absorption	(%)	<0.1
Elongation to fracture	(%)	2.4–3.1	Coefficient of linear thermal expansion	(K^−1^)	5.5 × 10^−7^
Softening temperature	(°C)	960	Specific heat capacity	(kJ∙kg^−1^·K^−1^)	0.86
Modulus of elasticity	(GPa)	89–110	Constant operating temperature	(°C)	680
Tensile strength	(MPa)	2800–4500	Melting temperature	(°C)	1450
Thermal conductivity	(W∙m^−1^·K^−1^)	1.67	Operating temperature range	(°C)	–260 to +750
Density	(kg·m^−3^)	2670	Coefficient of thermal conductivity	(W∙m^−1^·K^−1^)	0.031–0.038

**Table 5:** Consistency class according to slump-flow test (slump-flow test) [47].

Class	Slump-Flow SF ^a^ (mm)
SF1	550–650
SF2	660–750
SF3	760–850

^a^ specification cannot be applied for concretes with the aggregates characterised by *D*_max_ greater than 40 mm.

**Table 6:** Viscosity class in relation to time *t*_500_ [47].

Class	*t*_500_^a^, (s)
VS1	<2
VS2	≥2

^a^ specification cannot be applied for concretes with the aggregates characterised by *D*_max_ greater than 40 mm.

**Table 7:** Visual Stability Index (VSI) for the assessment of self-compacting mixtures [48].

VSI	Mixture Assessment	Criterion
0	Very stable	No visible segregation and no slurry leakage
1	Stable	No visible segregation, small slurry leakage
2	Unstable	Small segregation, large slurry leakage, small mortar leakage (film up to 10 mm)
3	Very unstable	Visible segregation, pile of aggregate in the centre of the mixture patch, large mortar leakage (over 10 mm), large slurry leakage

**Table 8:** Properties of the considered concrete mixtures.

Mixtures	Slump-Flow Consistency						Air Content (%)
	(Abrams Cone) SF (mm)	SF Class	Visual Stability Index VSI (%)	VSI Class	*t*_500_ (s)	Viscosity Class *t_500_*	
C5P0	750	SF2	unstable	2	2	VS2	2.6
C5P1b	660	SF2	stable	1	3	VS2	5.5
C5P50b50s	550	SF1	stable	1	4.3	VS2	6.0
C15P0	750	SF2	stable	1	2.5	VS2	3.2
C15P1b	680	SF2	stable	1	3.2	VS2	5.8
C15P50b50s	550	SF1	very stable	0	4.7	VS2	6.2

**Table 9:** Properties of the considered concretes.

Parameters	Unit	C5P0	C5P1b	C5P50b50s	C15P0	C15P1b	C15P50b50s
Volumetric density	(g·cm^−3^)	2.172	1.732	1.687	1.989	1.575	1.552
Open porosity	(%)	16.91	23.11	21.95	23.88	27.83	26.65
Water absorption by weight	(%)	6.58	11.27	12.31	10.64	14.25	15.57
Frost resistance: mass loss after 50 F-T cycles	(%)	2.1	0.03	2.8	7.88	1.39	2.02
Reduction in strength after 50 F-T cycles	(%)	1.3	0.4	4.0	8.1	3.2	9.2
Flexural tensile strength *f*_ct,flex_ after 28 days	(MPa)	9.56	7.02	10.04	8.36	5.53	8.96
Compressive strength *f*_c,cube#100_ after 7 days	(MPa)	67.28	21.85	42.67	50.66	11.64	35.84
Compressive strength *f*_c,cube#100_ after 28 days	(MPa)	74.63	31.38	54.03	68.32	26.23	52.77
